# Gene therapy for cystic fibrosis: new tools for precision medicine

**DOI:** 10.1186/s12967-021-03099-4

**Published:** 2021-10-30

**Authors:** Jin-A Lee, Alex Cho, Elena N. Huang, Yiming Xu, Henry Quach, Jim Hu, Amy P. Wong

**Affiliations:** 1 Program in Developmental and Stem Cell Biology, Hospital for Sick Children, 686 Bay Street, PGCRL 16-9420, Toronto, ON M5G0A4 Canada; 2 Department of Laboratory Medicine and Pathobiology, University of Toronto, Toronto, Canada; 3 Program in Translational Medicine, Hospital for Sick Children, Toronto, ON M5G0A4 Canada

**Keywords:** Cystic fibrosis, Stem cells, Lung, Organoids, CFTR, Alternative chloride channels, TMEM16A, Precision medicine

## Abstract

The discovery of the Cystic fibrosis (CF) gene in 1989 has paved the way for incredible progress in treating the disease such that the mean survival age of individuals living with CF is now ~58 years in Canada. Recent developments in gene targeting tools and new cell and animal models have re-ignited the search for a permanent genetic cure for all CF. In this review, we highlight some of the more recent gene therapy approaches as well as new models that will provide insight into personalized therapies for CF.

## Introduction

### Cystic fibrosis

Cystic fibrosis (CF) is the most common life-limiting fatal genetic disorder, affecting approximately 90,000 individuals worldwide [1]. It is an autosomal recessive disorder that requires mutations in the CF gene in both genetic alleles [2]. The CF gene encodes for a protein the cystic fibrosis transmembrance conductance regulator (CFTR) which is a protein chloride channel that belongs to the family of adenosine triphosphate (ATP)-binding cassette (ABC) transporters. It consists of two membrane-spanning domains (MSD1, MSD2), two nucleotide-binding domains (NBD1, NBD2) and the functional regulatory domain (R) with multiple phosphorylation consensus sites, which when phosphorylated, undergoes conformational change and opening of the chloride channel [3]. Mutations in the CF gene affecting CFTR expression, protein levels or function, now known as CFTR variants, affect multiple organ systems including the lung, pancreas, liver, gut and reproductive organs. Changes in chloride and bicarbonate transportation across this channel impairs epithelial cell functions including mucociliary transport of foreign agents out of the airways, elevated concentrations sweat chloride, impairment in pancreatic hormone regulation, and intestinal obstruction [4–6]. In the lungs, CFTR-mediated export of chloride and bicarbonate ions across the epithelium into airway surface liquid (ASL) plays a vital role in maintaining the ASL pH and airway secreted protein composition (i.e. mucins). Dehydration of the ASL thickens mucus secretions and impairs mucociliary clearance, antimicrobial enzyme activity and promotes a pro-inflammatory environment mediated by recurrent infections leading to lung damage [7].

### Classes of *CFTR* variants

**Fig. 1:**
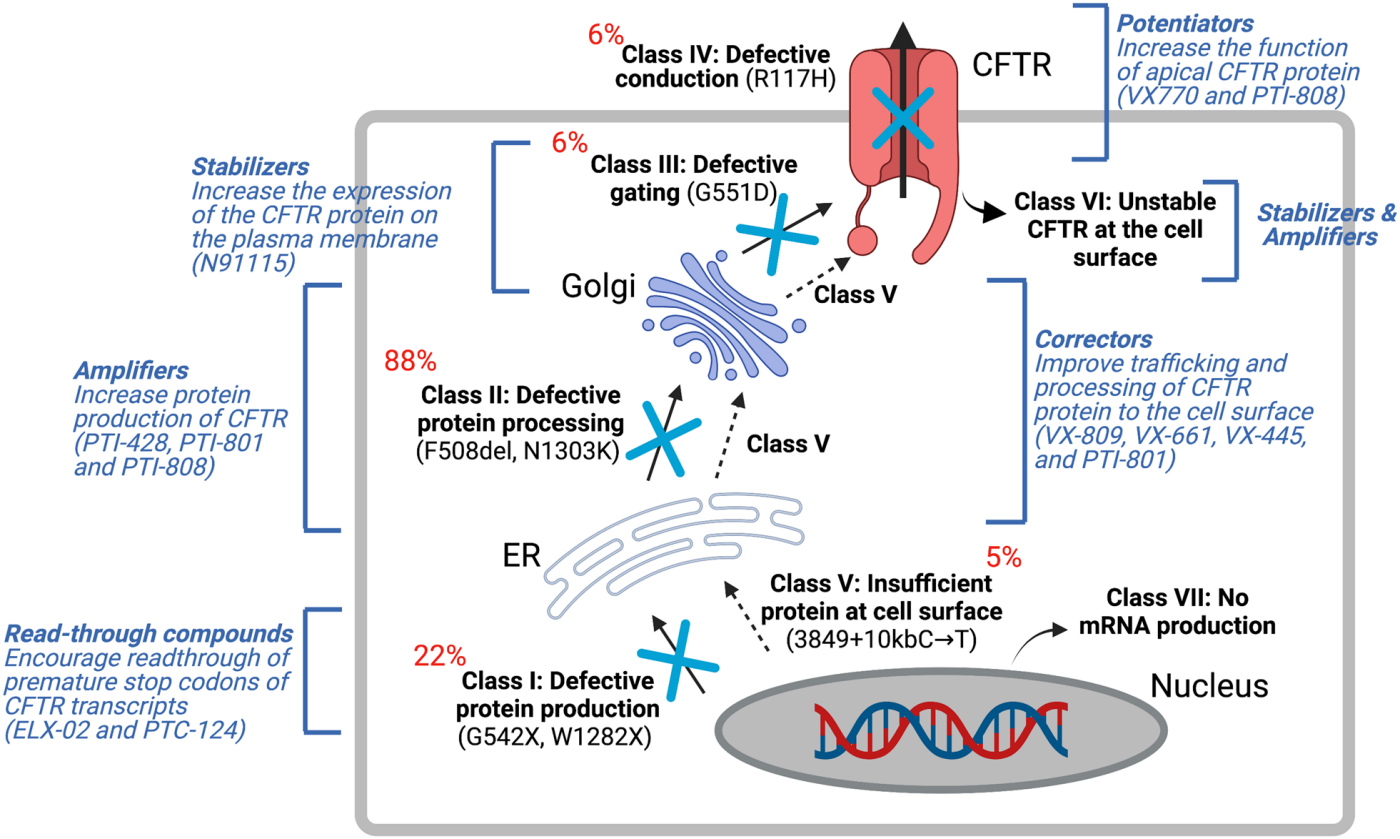
Classes of *CFTR* variants and CFTR modulators and the impact it has in CFTR expression and processing

In 1989, *CFTR* was identified and localized on the long arm of chromosome 7 (1q.31.2), consisting of 27 exons spanning about 215 kb of the genomic sequence [8–11]. While there have been > 2000 CF mutations identified to date (http://www.genet.sickkids.on.ca/cftr/), over 360 are CF disease-causing variants (www.cftr2.org). Recently, these variants have been categorized into 7 classes based on CFTR protein dysfunction and/or gene expression [12] (Fig. 1): Class I are protein production variants that result in no functional CFTR protein with roughly 22% of CF patients harboring at least 1 mutant allele; Class II are protein processing variants that create misfolded CFTR protein and reduced expression on the cell membrane to function. Approximately 88% of CF patients have at least 1 mutant allele and the main variant p.Phe508del (F508del)-CFTR caused by a mutational deletion of the amino acid phenylalanine at the position 508 of the protein; Class III are gating variants that impair CFTR gate opening and encompasses roughly 6% of CF patients; Class IV result in defective ion channel conduction and approximately 6% of CF patients harbour this variant; Class V are insufficient protein variants and results in a reduced amount of CFTR at the surface membrane capturing 5% of CF patients; Class VI affects the stability of CFTR that causes a reduction in membrane retention and 5% of CF patients harbor at least one allele of this variant, and finally Class VII affects *CFTR* mRNA expression resulting in no mRNA and includes large deletions such as the dele2,3(21 Kb) mutation.

### Advantages and disadvantages of *CFTR* modulator therapy

Over the past 30 years, tremendous advances in clinical interventions and CF research have allowed for transformative advances in CF therapy. Prior to the development of small molecules targeting the CFTR protein (CFTR modulators), treatment of CF was solely aimed at alleviating the symptoms associated with the disease [13]. In recent years, CFTR modulators capable of directly correcting the genetic defect are paving the way for a cure for CF [14]. Here, we briefly touch on some current CFTR modulators that have been approved or are currently in clinical trials.

CFTR modulators are classified into 4 groups (Fig. 1): correctors, potentiators, stabilizers and amplifiers. Small molecules aimed at stabilizing the misfolded protein in the cytosol to prevent degradation are known as correctors (examples include lumacaftor (VX-809), tezacaftor (VX-661), and elexacaftor (VX-445) from Vertex Pharmaceuticals and posenacaftor (PTI-801) from Proteostasis). Small molecules that bind to the NBD domain of the CFTR channel to facilitate its opening are known as potentiators, (examples include ivacaftor (VX770) and dirocaftor (PTI-808)). Stabilizers such as cavosonstat (N91115 from Nivalis) rescues the protein stability on the plasma membrane, promotes CFTR maturation and is currently in phase II clinical trials. Amplifiers increase the amount of CFTR production and include nesolicaftor (PTI-428), a current candidate in phase III clinical trials in combination with PTI-801 and PTI-808. Finally, for CF-causing variants where in-frame nonsense, frameshift, and splicing variants that introduce a premature termination codon (PTC) into the *CFTR* mRNA (i.e. W1282X and G542X), read through agents such as ELX-02 developed by Eloxx Pharmaceuticals and Ataluren PTC-124 by PTC Therapeutics were designed to restore functional protein production by overriding PTC signals [15]. However, early clinical trials currently underway for ELX-02 and PTC-124 failed to show significant improvement in FEV1 measurements in patients with at least 1 mutant allele in a phase III clinical trial [16]. The number of transcripts differ considerably depending on the site of the PTC, the cell type and the patient’s genetic background [15, 17]. Other small molecule inhibitors of the nonsense mediated decay (NMD) pathway such as SMG1 inhibitor (SMGi) can restore *CFTR* expression and function in cells harboring W1282X *CFTR* [18]. Therefore, combining small molecules to improve *CFTR* transcript production and/or stability with CFTR modulators may provide better clinical outcomes.

The approved CFTR modulator therapies ORKAMBI™ (a combination of VX-770 and VX-809) and SYMDEKO™ (a combination of VX-661 and VX-809) are combination treatments that has shown improved clinical benefits for some patients harboring F508del-*CFTR*. However, there are wide variations in responses to the drugs which suggest while the drugs may be used to treat the same genetic defect, other factors such as environmental [19–21] and gene modifiers [22–25] may influence therapy response. A recently approved drug, TRIKAFTA™ is a combination of 2 correctors (VX445 and VX661) and 1 potentiator (VX770) drugs that have shown incredible promise in improving lung function, sweat chloride conductance and lowering pulmonary exacerbations in F508del-CFTR individuals [26]. The short-term effectiveness of these modulators offer hope for restoring basic lung functions. However, the efficacy of this drug in effectively curing all CF individuals harboring at least 1 F508del allele remains unknown. Many rare CF variants are not eligible for current modulator treatment as these drugs are not expected to work such as for Class I production variants. Moreover, the long-term potential side effects of modulator treatment remain unclear [27] and with the costs for CFTR modulator therapy averaging over $300,000/year/patient [28], many CF individuals will not receive potential life-saving therapies without financial support or reimbursements. Therefore, new therapy approaches are still needed to treat all CF.

### Gene therapy approaches for CF

Gene therapy offers great hope for the treatment of genetic diseases/disorders. By replacing the genetic mutation with a “correct version” of the *CFTR* gene, this method offers a potentially permanent cure. Indeed, since the discovery of the CF gene, many studies have attempted to correct the *CFTR* mutations through gene therapy approaches. While gene correction showed limited success in both cell and animal models [29–31], therapy for patients had proven to be more difficult. In-vitro studies have suggested that not all cells need to express normal CFTR to effect normal epithelial functions. In a mixing experiment where normal cells were mixed with CF mutant cells, only 6–10% of the epithelium needed to contain epithelial cells expressing normal CFTR to restore chloride transport similar to normal epithelia [32]. Conversely, in a gene targeting study, up to 25% gene correction could restore mucus transport in homozygous F508del human airway epithelial cells [33]. The number of cells harboring wild-type *CFTR* that is needed to translate into clinical benefit in patience remains unknown. However, theoretically correcting a stem cell population within the airways may provide a renewable and long-term source of endogenous cells capable of renewing the damaged epithelia with cells that express wild-type *CFTR*. Yet surprisingly, with the exception of a Phase I and II clinical trial for MRT5005 [https://www.cff.org/Trials/Pipeline/details/10157/MRT5005], a drug that delivers *CFTR*-encoded mRNA to the lungs (RESTORE-CF), there are no other clinical trials for CF gene therapy. This may largely be due to several reasons: 1. The need for repeated delivery due to the inability to target stem/progenitor cells of the airways to sustain expression during cell turnover, 2. Suboptimal delivery or low efficiency of targeting of the donor plasmid/gene to the CF airways due to the highly inflammatory microenvironment, 3. The inability to deliver large DNA fragments of the *CFTR* gene effectively with current delivery methods, 4. Concerns of off-target safety that can result in insertional mutagenesis, and 5. Immune barriers limiting effective delivery of viral vectors. In this review, we briefly touch on some of the more recent genetic approaches that can rejuvenate CF gene therapy and touch on new cell and animal models that are enabling the testing of current gene targeting strategies and providing insight into personalized approaches for CF therapy.

#### Gene editing approaches

Gene editing tools can provide new gene therapy strategies to achieve permanent correction. Here we list a few editing tools used to date to test the efficacy of genetic correction for CF in-vitro.

### Zinc Finger Nucleases (ZFNs) and transcription activator-like effector nucleases (TALEN)

**Fig. 2:**
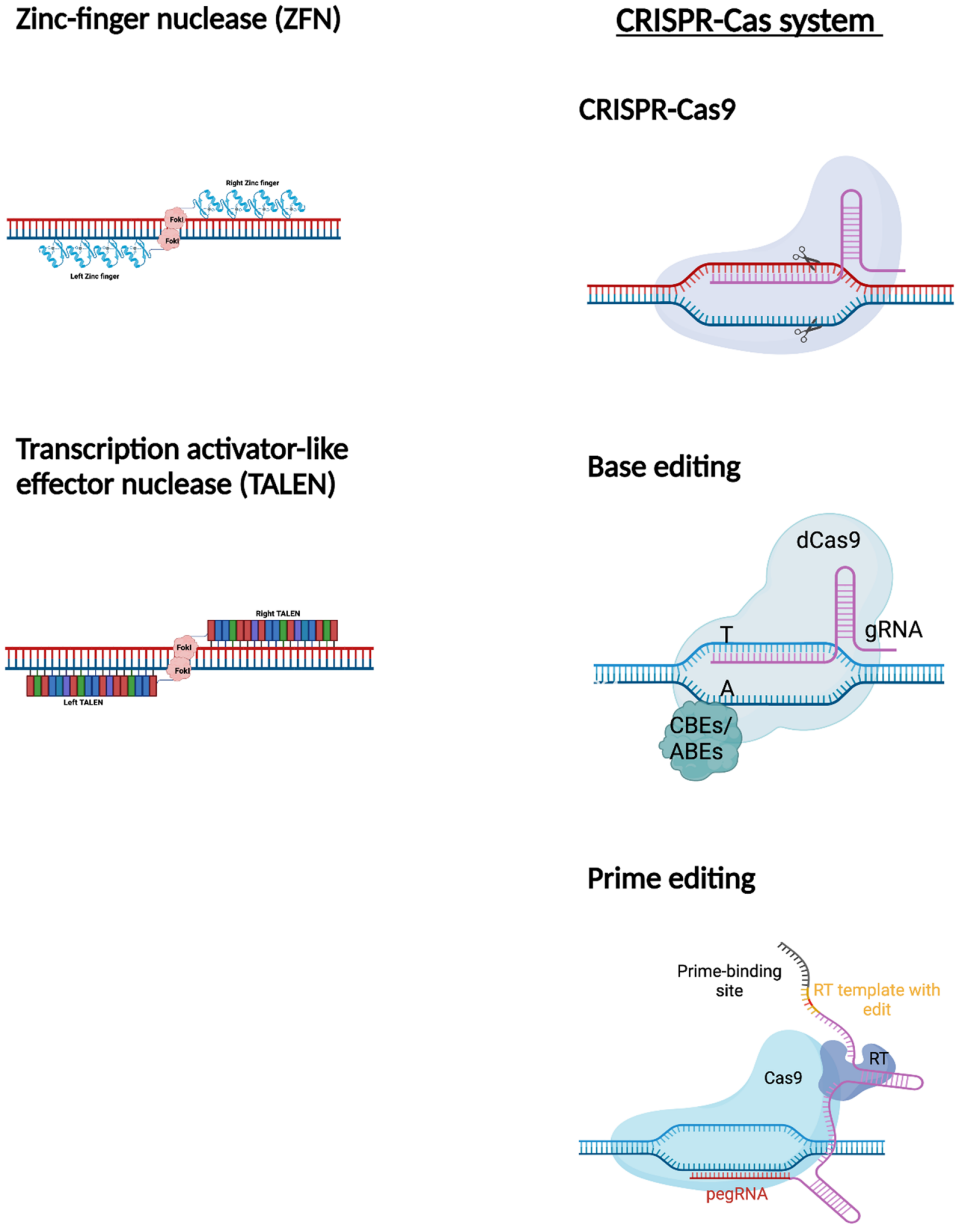
Graphics of gene editing technologies

Early developments of gene editing approaches included use of artificial restriction enzymes, Zinc Finger Nucleases (ZFNs) and transcription activator-like effector nucleases (TALEN) [34, 35], (Table 1; Fig. 2). These gene modification tools enabled precise genome editing through targeted nucleases cleavages and renewed hope for gene therapy. ZFNs are composed of specific pairs of oligos attached to a FokI restriction enzyme that facilitate a precise double-strand break (DSB) at the target site [36]. TALENs are composed of TALE repeats that bind and recognize extended DNA sequences and are also attached with a FoKI restriction enzyme to create a DSB [37, 38]. In both instances, the DSB induces DNA repair mechanisms by either non homologous end joining (NHEJ), or homology-directed repair (HDR) [39, 40]. Neither ZFN and TALENs technology have been used in CF gene therapies and in the advent of CRISPR-Cas systems, gene editing using the latter tool is more flexible making it the editing tool of choice for many researchers. The specific requirement of a pair of ZFNs reduces the number of target sites that can be identified for gene correction. Moreover, the low binding affinity of the ZFN creates undesirable off-target mutations in the genome [41]. TALEN has shown less off-target and better binding affinity than ZFN, however, the size for cDNA encoding a TALEN (3 kb) can be an issue for delivery into cells with a limited cargo size [42].

**Table 1:** Advantages and disadvantages of gene editing tools

	ZFN	TALEN	CRISPR/Cas9	Base Editing	Prime editing
Mechanism	Type IIs restriction enzyme, FokI endonuclease, fused to pair of ZFN DNA binding domains Recognize 18-36 bp of DNA sequence Target DNA sequence break by protein-DNA interaction	Type IIs restriction enzyme, FokI endonuclease, fused to pair of TALEN DNA binding domains Recognize 30-40 bp of DNA sequence Target DNA sequence break by protein-DNA interaction	Few Cas endonuclease options for broader specificity and flexibility (Cas9, Cas12) PAM sequence require to design sgRNA Target DNA sequence break by DNA-RNA interaction	Direct conversion of a DNA base to another without DSBs at a target locus Permanent conversion of C-G to T-A base pairs by cytosin base editor (CBEs) Enzymatically convert A-T base pairs into G-C base pairs by adenine base editors (ABEs)	Fusion complex composed of a catalytically impaired Cas9 protein and an engineered reverse transcriptase Can recognize DNA of any sequence size
Efficiency	Low	Low	High	High	High
Advantages	Currently being used in clinical trials for HIV and Hunter’s syndrome Low immunity and Small protein size	Target any DNA sequence Less cytotoxic effects	Highly predictable target sequence Easy to design and possible to target only 1 bp of target sequence Potentially target multiple genes simultaneously	No random insertion and deletions because do not require DNA break High A > G and C > T conversion	No random insertion and deletions because do not require DNA break Can be used to generate different mutation types (insertions, deletions, and point mutations)
Limitations	Difficult due to extensive cloning needed to link two zinc finger modules together and expensive to design	Sensitive to DNA methylation Require pair of TALEN with two independent DNA binding sites	Require PAM site near the target DNA sequence to design gRNA Off-target effect observed Cas9 protein too large for AAV-based delivery	Only accounts for 4 out of 12 possible base-to-base conversions Too large for AAV-based delivery Difficult to edit DNA sequence that several A or C residues are nearby	High targeting efficiency but may depend on cell type Too large for AAV-based delivery Detection of undesired off-target effects and on-target mutation

### CRISPR gene editing

In 2013, a new gene-editing tool used by bacteria to fend off bacteriophages by called clustered regularly interspaced short palindromic repeats (CRISPR) and it’s enzyme CRISPR associated protein 9 (Cas9) [43] was shown to be useful in editing the genomes of cultured mammalian cells [44]. The precise editing of the CRISPR-Cas9 system along with the versatile use of the system to silence genes by removing part of the gene or substituting the gene with desired ones has made the CRISPR-Cas9 system the preferred editing tool for gene editing. Moreover, the relative ease in designing a specific target site and low cost allows efficient gene editing to be done within a relatively short period of time [45]. The CRISPR-Cas9 is composed of two main modules: the guide RNA (gRNA), and the Cas9 protein enzyme. The gRNA is designed to recognize a specific sequence motif near the target site and recruits the Cas9 protein to cut and create a double-stranded DNA break (DSB). The cell’s natural DNA repair mechanisms are then activated to repair the cleaved DNA through NHEJ or HDR [39, 40]. NHEJ directly ligates the broken ends, and can create “indels” or insertion or deletions of genes effectively creating mutants [46]. However, with a repair template, the HDR response will enable homologous recombination. This method is useful for introducing a desired gene (or a wild-type version of a gene). However, the frequency of HDR is very low [47] and therefore efficiency of “repairing” or replacing a mutant gene remains a challenge.

*(i) Base editing: *The CRIPSR-Cas9 system’s classical reliance on introducing DSBs poses an efficiency problem since undesirable random insertions or deletions (indels) occur more often at DNA cleavage sites than HDR. Base editing was thus pioneered to increase the efficiency of the CRISPR-Cas9 system by circumventing the need for DSBs altogether, allowing for the direct conversion of a DNA base to another without DSBs at a target locus [48]. Cytosine base editors (CBEs) facilitate the permanent conversion of C-G to T-A base pairs, while adenine base editors (ABEs) enzymatically convert A-T base pairs into G-C base pairs [49]. In the contexts of CF, base editing could then be an attractive new tool in treating CF, since many *CFTR* variants could be rescued with a single base pair change. Accordingly, Geurts et al. recently provided support to the efficacy and feasibility of utilizing such base editing tools safely within human cells to potentially treat CF with two respective ABEs [50]. A caveat of base editing is the limitation of only 4 possible base-to-base conversions and is too large for certain gene delivery vectors.

*(ii) Prime editing*: Prime editing has recently become an attractive advancement in the CRISPR toolbox [51]. This gene editing technology makes it possible to edit a specified DNA sequence, of variable lengths at a target site, with a fusion complex composed of a catalytically impaired Cas9 protein and an engineered reverse transcriptase [51]. A prime editing guide RNA (pegRNA) encodes the desired gene edit and directs the fusion complex to the target site [51]. As a possible gene replacement therapeutic technology, prime editing is very promising in the context of CF, given the most common *CFTR* variant (CFTR-F508del) has been repaired by prime editing in patient-derived intestinal organoids [52]. However, prime editing did result in varying degrees of targeting efficiency and undesired off-target mutations were also observed [52]. Nevertheless, since the *CFTR* gene is large, and a complete replacement of a mutant gene with wild-type *CFTR* would likely be inefficient, prime editing is leading method to address the vast number of CF disease-causing variants.

### Gene delivery

There are several gene delivery methods to introduce a therapeutic gene or gene targeting. Both non-viral and viral delivery vectors have been tested in CF gene therapy research.

*(i) Non-viral vectors: *Non-viral vectors were developed as a strategy to deliver the *CFTR* gene. These non-integrating gene delivery methods do not disrupt the host genome and thus the risk of causing mutagenesis are low. Non-viral vectors are not restricted in the cargo load enabling larger donor DNA fragments to be used for gene repair. However, the efficacy of gene delivery is comparatively lower than viral methods. To enhance gene transfer into the nucleus, a cationic lipid is used to formulate the plasmid DNA [53] complexed with *CFTR* enhanced chloride transport by 20% in CF patients compared to non-CF levels [54]. Using a nebulized cationic lipid pGM169/GL67A to deliver the donor DNA, up to 3.7% increase in CFTR function in the lungs of CF patients was observed [55, 56]. The drawback of the cationic liposome-mediated approach is the need for repeated delivery as transient expression of *CFTR* did not have a lasting effect [57]. Despite these efforts, non-viral based methods of gene delivery cannot permanently restore lung functions.

*(ii) Viral vectors*: To improve efficacy of targeting the cells, several viral based delivery methods have been tested to including adenovirus (Ad), adeno-associated virus (AAV), and retroviral vector in pre-clinical and clinical trials to deliver the corrected *CFTR* gene.

#### Adenovirus (Ad)

Based vectors were once the preferred delivery vectors for gene delivery [58, 59]. Mutational deletion of viral replication genes and host immune cell evasion genes early region 1 and 3 (E1/E3) respectively, removed the ability of the virus to self-replicate making these viral vectors attractive for gene therapy. However, leaky expression of viral genes from E1 deleted vectors, in addition to capsid proteins, could elicit host immune responses to the Ad vectors [60–62]. The first clinical trial (in 1993) for CF gene therapy using an adenovirus vector failed to restore *CFTR* expression in CF patient’s nasal epithelia [63, 64]. This led to the identification and testing of other adenovirus serotypes 2 and 5 in CF clinical trials which resulted in transient restoration of chloride transport in the nasal and bronchial epithelium [65, 66]. However, evidence of a pro-inflammatory response was found with these Ad vectors which required repeated administration for effective gene delivery [63, 65]. Even so, the trials have only demonstrated limited clinical benefits in CF patients [66].

#### Adeno-associated virus

AAV-based vectors have been tested as another gene delivery tool. With the ability to transduce terminally differentiated and non-dividing cells, AAV can also persist longer *in-vivo* [67] compared to its Ad counterpart. Transient immunosuppression can improve re-administration of AAV vectors in mouse lungs up to 8 months [68]. In 1998, the first successful human clinical trial with repeated delivery of AAV2-CFTR into the maxillary sinuses [69] demonstrated restoration of CFTR function without noticeable toxicity or an elevated immune response after 2 weeks of delivery. However, other clinical trial studies performed years later failed to show sufficient CFTR functional correction by AAV-CFTR [70, 71]. One caveat of the AAV vectors is the limited target gene size (less than 4.6 kb) that can be inserted into the viral vector for efficient expression.

#### Helper-dependent adenoviruses (Hd-Ad)

To avoid the harmful immune response of Ad, the Helper-dependent Adenovirus (Hd-Ad) was developed [72]. Deletion of all viral coding sequences allows Hd-Ad to deliver large DNA cargo (to 37 kb) without eliciting host immune responses [73, 74]. One unique feature of the Hd-Ad vectors is that they can be used to deliver both a gene editing endonuclease system and donor DNA in a single vector to achieve site-specific gene integration without expressing the endonuclease following gene correction [75–77]. Gene correction using Hd-Ad in CF mouse and pig airway basal cells can restore CFTR function similar to levels observed in normal wild-type cells as measured by fluorescence imaging plate reader (FLiPR) assay [30, 72, 78–81]. HD-Ad vectors have also been shown to be effective in correcting the *CFTR* gene in the lungs of CF knockout mice [82]. However, a major challenge remains for *in-vivo* gene therapy as the ability to sustain therapeutic effects is lost due to airway cell turnover. Therefore, targeting a stem cell compartment within the airways has become an attractive goal for permanent CF gene correction.

#### Retroviruses and lentiviruses

Retroviral and lentiviral vectors have been used for gene delivery methods as early as the late 1990s. Retroviruses harboring human *CFTR* gene transduced into rabbit tracheal epithelial cells showed persistent expression in the airways for up to 3 weeks. However, the transduced capacity by retroviruses were low and transduction occurred only in wounded areas [83]. Lentiviral vectors have been effective in delivering *CFTR* transgene into the airway epithelium [84] with potential to target the lung stem cell population for sustained and persistent *CFTR* expression [85]. While both retroviruses and lentiviruses can efficiently target host cells and integrate into the host genome, there remains significant concerns over their use as a delivery vector for gene therapy. The host immune responses remain a significant barrier in efficacious delivery of exogenous genetic materials by viral methods. In the context of CF airway disease, the proinflammatory milieu of the diseased airways compounded by the mucosal obstructions poses a challenge for any gene delivery methods. Second, there are concerns of insertional mutagenesis, epigenetic silencing, and secondary impact of altered expression levels derived by using viral promoters to drive the un-regulated expression of the transgene [86, 87].

Therefore, while new gene editing approaches may increase the targeting efficiency of gene correction, precise and efficient delivery of the genetic tools to the right cell type for permanent gene correction remains a barrier to clinical use. To study this, new animal and advanced stem cell-based models may enable research into cell delivery and targeting strategies.

#### Animal models of CF

**Fig. 3:**
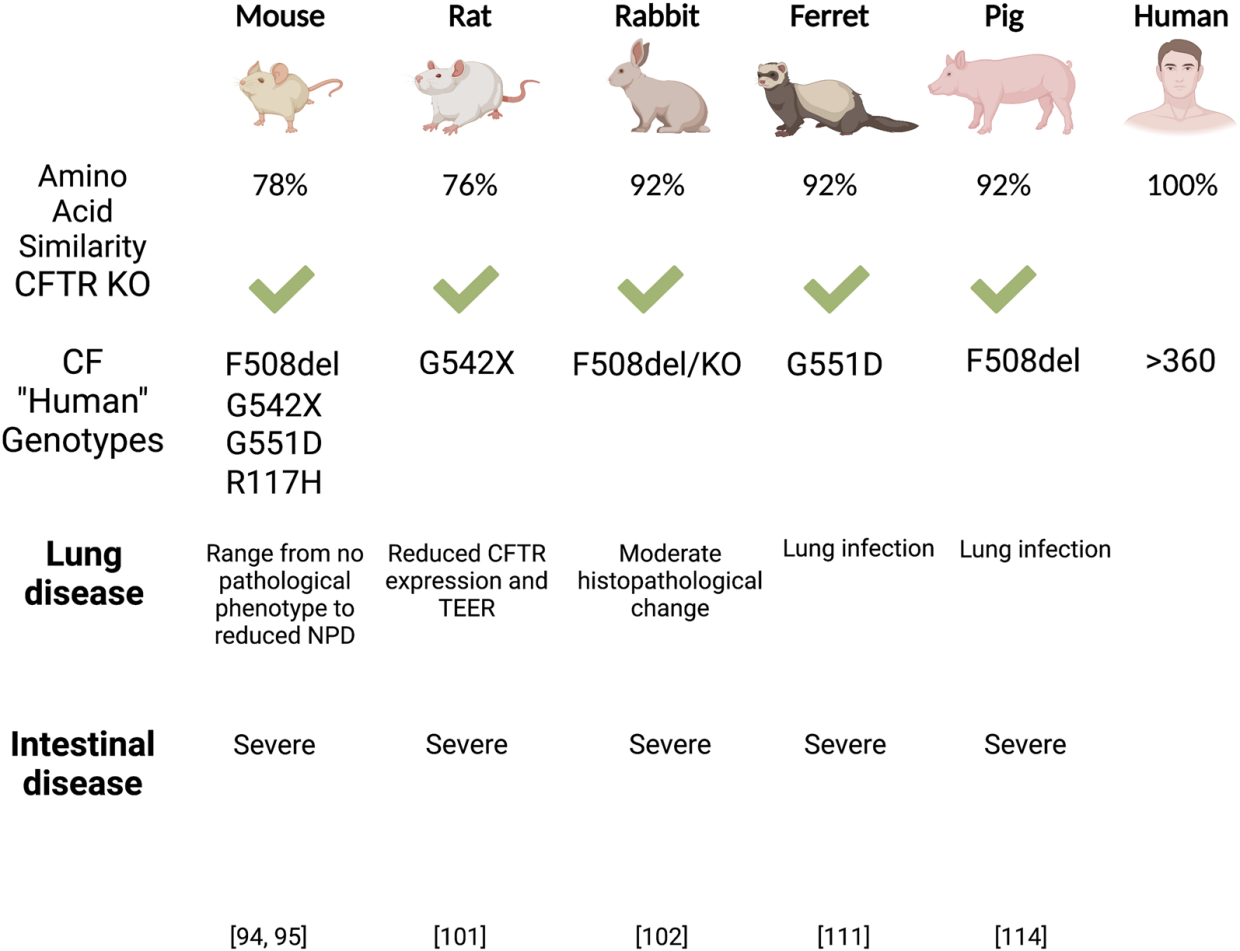
CF animal models compared to human disease phenotypes

Animal models of CF are valuable tools that may be utilized to further understand disease pathogenesis and test new therapeutics. There are two fundamental issues that remain to be resolved before gene therapy can become viable for patients, and animal models provide a relevant platform through which these obstacles may be safely addressed. First, in-vivo efficiencies of gene targeting need to achieve a level that will translate to therapeutic outcome. Second, the efficacy of gene targeting must outweigh concerns of off-target mutagenesis from the gene editing tools. Animal models have traditionally been useful models to understand basic mechanisms of disease pathogenesis. Recent animal models for CF, especially those harboring human CF variants offer opportunities to test new emerging CFTR modulators for which these modulators are designed to specifically target the specific functional outcome. Here we briefly touch on several of these animal models and their use in CF therapy discovery (Fig. 3).

*(i) Mouse model*: With a 78% amino acid sequence conservation between mouse and human *CFTR* (h*CFTR*) [88], the use of mice for disease modelling comes as no surprise when also considering practical factors like costs, breeding time, and ease of maintenance. However, CF mouse models only exhibit mild pancreatic disease [89, 90] if any, present variable gallbladder abnormalities [90–92], and liver pathologies are largely only observed in mice studied later in life [89]. While new humanized mouse models have become available, and can be used to study CFTR modulator efficacies, they possess a major limitation in harboring ~ 6 copies of the h*CFTR* gene [93–95]. Therefore, it remains unclear how effective these humanized models are for gene therapy testing but may be a good model for CFTR modulator testing.

*(ii) Rat model*: CF rat models present similar phenotypes with CF mice. Like the CF mice, the rat models do not recapitulate spontaneous lung infection or pancreatic and liver disease [96, 97] though some models have displayed exocrine pancreas histopathology [98]. Nevertheless, rats possess a 76% amino acid sequence identity to h*CFTR* [99] and have submucosal glands in the large airways [97, 100]. Rat models have also provided the groundwork for exploring new genetic advancements in CF modelling, like the generation of the first G542X CF nonsense mutation rat model with CRISPR-Cas9 [101], and a new F508del rat models that may be invaluable in the development of therapeutics [97].

*(iii) Rabbit model*: Rabbit models of CF are rather new to the field [102, 103] thus the relevance to human CF disease remains to be seen. However, rabbits present as a very promising model for the study of lung diseases in general, due to their airway anatomy and inflammatory responses [104]. Further, there is a 92% amino acid sequence conservation between rabbit and human *CFTR* [88]. A caveat of the rabbit model is they lack submucosal glands within their airways [100, 104] which contain CFTR-expressing cells in human airways.

*(iv) Ferret model*: Due to the highly conserved anatomy between human and ferret lungs [105, 106], ferret CF models accurately mirror the key disease phenotypes of CF, including those unable to be recapitulated in other models [107–109] With a sequence homology of 92% with h*CFTR* [88], and an abundance of submucosal glands throughout their airways [110], ferrets are an attractive translational model of CF [111]. A caveat of the ferret model is the costs associated with maintaining these animal colonies and current CF ferret models require CFTR modulators to survive, making long-term study of the disease pathogenesis difficult.

*(v) Pig model*: Pig models share a 92% amino acid sequence identity with h*CFTR* [88], and arguably offer the highest translational potential for CF research due to their comparable genetics, physiology, and anatomy to humans [112–114]. However, porcine CF models present an even larger practical and cost challenge than ferrets. Their sheer size, while beneficially comparable to humans, calls for much consideration regarding labor costs and maintenance. For testing new drugs, the pig model can become astronomically expensive. Nevertheless, CF pig models recapitulate all key CF disease phenotypes, though notably with more severe manifestations than in humans [113–117].

### Cell models for studying CF disease pathogenesis and therapy.

**Fig. 4:**
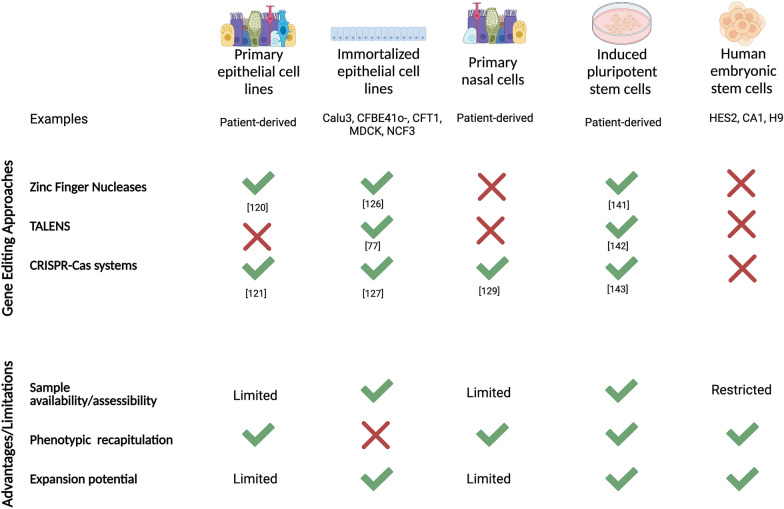
Cell models to study CF disease and therapies. For gene editing approaches, green “✓” indicates research data supporting the use of these approaches in the cell models for CF gene correction. Red “X” indicates no information available. For advantages/limitations section, green “✓” indicates possible and red “X” indicates not possible

*(i) Current gold-standard lung cell models*: Cell models have played instrumental roles in understanding the biophysical properties of CFTR, the mechanistic cause of the defects and evaluating novel therapeutic strategies (Fig. 4). Human primary epithelial cell lines have been the main tool for assessing ion channel functions and for drug development [118–121]. While recent improvements in culture conditions have improved the expansion potential of primary cells, this expansive ability is limited [122] and primary cells enter senescence shortly in culture. To circumvent this, immortalized epithelial cell lines, such as A549, BEAS-2B, Calu-3 and 16HBE14o, are commonly used to study drug transport, metabolism, and epithelial integrity [123–127]. However, these immortalized cell lines are derived from lung tumour cells or have been transformed, and thus do not show original lung cell characteristics or reflect the repertoire of epithelial cell types found in the native lungs. Primary nasal cells are an alternative cell type to study CF airway disease due to the ease of generating nasal epithelial cultures from patients. The pros of these cells are the relative ease of obtaining samples from patients and they can be sampled several times (if needed). Studies have suggested nasal epithelial cells are a good surrogate of airway bronchial epithelial cells [128, 129]. However, like primary bronchial cells, the ability to expand these cells in culture for sufficient use without re-sampling remains a problem. In addition, sampling variability can impact CFTR protein expression and function of the epithelium. Recently, lung stem cells isolated from bronchoalveolar lavage fluid can generate renewable airway organoids for multiple passages in cultures [130]. It remains to be seen whether a method of airway organoid generation can be achieved from individuals with airway diseases for disease modeling. Nonetheless, generation of a renewable source of patient-specific lung airway cells is a key enabler for identifying patient-specific therapies for lung diseases.

*(ii) Human pluripotent stem cell (PSC) models for personalized medicine*: Human embryonic stem (hES) cells were discovered in 1998 and hold enormous promise to repair disease and regenerate tissues [131]. With the ability to self-renew and differentiate into cells of all three embryonic germ layers endoderm, ectoderm and mesoderm, hES became an intriguing source of cells for regenerative medicine. However, research in the use of hES for regeneration faced paucity due to the growing ethical concerns associated with the use of “embryonic/fetal” tissue. In 2006, the first discovery of induced pluripotent stem cells (iPSC) was made and revealed these cells shared similar characteristics to mouse ES [132]. By 2007, the first human iPSC was made by introducing four transcription factors associated with pluripotency to fibroblasts [133]. Since this discovery, therapeutic applications of human iPSC have led to > 65 market competitors offering iPSC-based products. Indeed, iPSC are a great source of cells for patient-specific disease modeling, drug discovery and personalized regenerative medicine. Biobanks of iPSC from individuals with various genetic mutations have become a useful resource for disease modeling. The Hospital for Sick Children in Toronto has now acquired over 100 CF patient cells harboring various *CFTR* variants and generated iPSC from each individual including some gene-corrected isogenic iPSC lines for benchmarking patient-specific “normal” responses [134]. This will undoubtedly enable research in modelling CF organ and patient-specific disease and therapy discoveries.

Differentiation of human iPSC into multiple tissue cell types has now been achieved albeit with varying efficiencies. Most directed differentiation methods use a stepwise approach of activating and/or inhibiting pathways known to affect developmental growth in animal models, especially the mouse. Indeed, we and others have identified key developmental pathways required to generate lung epithelial cells from human iPSC [135–138]. Moreover, airway and intestinal cells derived from homozygous F508del CF iPSC model CF phenotype (lack of CFTR membrane expression) can be used to screen for CFTR small molecule correctors [136, 139, 140]. We have shown that CF iPSC-derived airway cells are amenable to high throughput CFTR functional screens—a step towards using these cells for personalized medicine [139–143]. Recently, we have improved the generation of lung cells from human PSC and demonstrate the utility of capturing *CFTR* expression and function in the differentiated cells modeling development [136, 144]. Understanding the impact of mutant CFTR during development remains poorly understood and these new PSC models will advance our understanding of the prenatal origins of disease mechanisms.

Another benefit of using iPSC models is the ability to determine both patient and tissue-specific responses. This is important as *CFTR* expression and activity levels differ in different tissues. Correction of CF mutations have been tested in iPSC, however the efficacy of these gene-editing strategies in-vivo remains to be seen [141–143]. Ultimately, establishing predictive patient and tissue specific models to predict patient outcome is key to advancing precision medicine.

### New models, new gene editing tools, new targets?

One of the biggest challenges in generating treatment strategies for CF is the sheer number of CF-causing variants. Even among patients with the same variant, there are vast differences in severity of symptoms and responses to treatments. To date, treatment options for CF are mutation-dependent, and no viable options exist to universally address all CF patients. Though recent advancements in gene editing have fostered hope for personalized treatments, this is neither viable nor practical for treating all CF.

Recently, Kemaladewi et al. demonstrated a novel mutant-independent therapeutic approach to treat congenital muscular dystrophy type 1A (MDC1A) [145]. Using CRISPR, the feasibility of treating inherited diseases by looking beyond the singular disease-causing gene, and instead targeting compensatory modifier genes, was illustrated. In the context of CF, ion channels aside from CFTR have been implicated in CF disease severity and responses to modulator therapy. Therefore, targeting other ion channels known to also affect CF disease severity such as the sodium channel ENaC [146] or alternative ion channels TMEM16A (*ANO1* [147, 148]) and SLC26A9 [149, 150] may need to be assessed to find effective therapies for all individuals with CF.

## Conclusion

Since the discovery of the CF gene over 30 years ago, it has become apparent that finding an effective therapy to treat all CF remains a challenge. While the discoveries of new small molecule modulators have greatly advanced treatment for some CF, the effectiveness of these lifesaving drugs have not been universally effective and rather limited to specific classes of mutations. Rare *CFTR* variants remain uncured. Now, with recent advances in new gene editing tools coupled with both iPSC-derived tissue models and new animal models, new precise gene targeting methods to treat CF disease will emerge and lead to potential effective personalized therapies. Classical approaches of targeting the disease-causing variant may also be replaced or coupled with mutation-agnostic approaches to treat complex CF phenotypes and with improved pre-clinical models, this can now be tested. With new advancements in gene editing technologies coupled with advanced cell models to test gene engineering approaches, this will lead to rapid developments of new therapies for all CF.

## Data Availability

Not applicable.

## Abbreviations

CF: Cystic fibrosis
CFTR: Cystic fibrosis transmembrane conductance regulator
F598del: P.Phe508del
ABC transporters: Adenosine triphosphate (ATP)-binding cassette transporters
MSD1 and MSD2: Membrane-spanning domain 1 and 2
NBD1 and MBD2: Nucleotide-binding domain 1 and 2
R domain: Regulatory domain
ASL: Airway surface liquid
PTC: Premature termination codon
NMD: Nonsense mediated decay
ZFNs: Zinc Finger Nucleases
TALEN: Transcription activator-like effector nucleases
DSB: Double-strand break
NHEJ: Non homologous end joining
HDR: Homology-directed repair
CRISPR: Clustered regularly interspaced short palindromic repeats
Cas9: CRISPR associated protein 9
gRNA: Guide RNA
indels: Insertions or deletions
CBEs: Cytosine base editors
ABEs: Adenine base editors
pegRNA: Prime editing guide RNA
Ad: Adenovirus
AAV: Adeno-associated virus
Ad: Adenovirus
E1: Early region 1
E3: Early region 3
AAV: Adeno-associated virus
Hd-Ad: Helper-dependent adenoviruses
FLIPR: Fluorescence imaging plate reader
h*CFTR*: Human cystic fibrosis transmembrane conductance regulator
hES: Human embryonic stem
iPSC: Induced pluripotent stem cells
MDC1A: Muscular dystrophy type 1A
ENaC: Epithelial sodium channel
TMEM16A: Transmembrane member 16A
ANO1: Anoctamin-1
SLC26A9: Solute carrier family 26 member 9
